# On Absorption Modeling and Food Effect Prediction of Rivaroxaban, a BCS II Drug Orally Administered as an Immediate-Release Tablet

**DOI:** 10.3390/pharmaceutics13020283

**Published:** 2021-02-20

**Authors:** Varun Kushwah, Sumit Arora, Miklós Tamás Katona, Dattatray Modhave, Eleonore Fröhlich, Amrit Paudel

**Affiliations:** 1 Research Center Pharmaceutical Engineering (RCPE) GmbH, Inffeldgasse 13, 8010 Graz, Austria; varun.kushwah@rcpe.at (V.K.); sumit0607@gmail.com (S.A.); datta.niper@gmail.com (D.M.); eleonore.froehlich@medunigraz.at (E.F.); 2 Simcyp Division, Certara UK Limited, Level 2-Acero, Sheffield S1 2BJ, UK; 3 Department of Pharmaceutical Chemistry, Semmelweis University, Hőgyes Endre u. 9., H-1092 Budapest, Hungary; Katona.Miklos@egis.hu; 4 Galapagos, Analytical Development CMC, Generaal De Wittelaan L11 A3, 2800 Mechelen, Belgium; 5 Center for Medical Research, Medical University of Graz, Stiftingtalstr. 24, 8010 Graz, Austria; 6 Institute for Process and Particle Engineering, Graz University of Technology, Inffeldgasse 13, 8010 Graz, Austria

**Keywords:** in vitro–in vivo correlation, physiologically based pharmacokinetic model, BCS Class II, Rivaroxaban, Xarelto, food effect, population kinetics

## Abstract

The present work evaluates the food effect on the absorption of rivaroxaban (Riva), a BCS II drug, from the orally administered commercial immediate-release tablet (Xarelto IR) using physiologically based pharmacokinetic (PBPK) and conventional in vitro–in vivo correlation (IVIVC) models. The bioavailability of Riva upon oral administration of Xarelto IR tablet is reported to exhibit a positive food effect. The PBPK model for Riva was developed and verified using the previously reported in vivo data for oral solution (5 and 10 mg) and Xarelto IR tablet (5 and 10 mg dose strength). Once the PBPK model was established, the in vivo performance of the tablet formulation with the higher dose strength (Xarelto IR tablet 20 mg in fasted and fed state) was predicted using the experimentally obtained data of in vitro permeability, biorelevant solubility and in vitro dynamic dissolution data using United States Pharmacopeia (USP) IV flow-through cell apparatus. In addition, the mathematical IVIVC model was developed using the in vitro dissolution and in vivo profile of 20 mg strength Xarelto IR tablet in fasted condition. Using the developed IVIVC model, the pharmacokinetic (PK) profile of the Xarelto IR tablet in fed condition was predicted and compared with the PK parameters obtained via the PBPK model. A virtual in vivo PK study was designed using a single-dose, 3-treatment cross-over trial in 50 subjects to predict the PK profile of the Xarelto® IR tablet in the fed state. Overall, the results obtained from the IVIVC model were found to be comparable with those from the PBPK model. The outcome from both models pointed to the positive food effect on the in vivo profile of the Riva. The developed models thus can be effectively extended to establish bioequivalence for the marketed and novel complex formulations of Riva such as amorphous solid dispersions.

## 1. Introduction

Developing and deploying approaches that enable predicting the in vivo efficacy and safety profile of pharmaceutical drug products enormously expedite the product and process development effort as well as reduce the need for expensive clinical studies. For solid oral dosages, a thorough biopharmaceutical characterization at the in vitro level, such as solubility and dissolution testing using biorelevant media, study of food–formulation interaction, in vitro membrane permeability and drug transport studies etc., provides the input data for in vivo absorption prediction. In recent years, prominent progress has been made in the in vitro biopharmaceutics profiling as well as in silico modeling for solid drug products [1]. A set of biorelevant and clinically relevant in silico models are expected to account for the critical formulation and physiological factors to facilitate the correlation between in vitro drug dissolution and in vivo pharmacokinetic profiles.

A widely accepted approach to assess the correlation between in vitro dissolution and in vivo bioavailability of an immediate-release (IR) drug product is based on the Biopharmaceutics Classification System (BCS). The BCS categorizes drug substances into one of four classes based on their solubility and permeability. In general, BCS Class I (highly soluble and highly permeable) drugs are well-absorbed. The rate-limiting step to absorption is dissolution or gastric emptying. In vitro–in vivo correlation (IVIVC) is expected if dissolution rate is slower than gastric emptying rate. In the case of IR drug products containing BCS Class II drug substance (dissolution as a rate-limiting step for absorption), conventional IVIVC can be used to establish the (cor)relation between the in vitro drug release and in vivo plasma concentration. Dissolution media and methods that reflect the in vivo controlling process are particularly important in this case if good in vitro–in vivo correlations are to be obtained. For BCS Class III drugs (for which permeability is the rate-limiting step for absorption), limited or no IVIV correlation is expected with the dissolution rate. Drug products containing BCS Class IV drug substance (low solubility and low permeability) has to be evaluated case by case; however, these drugs exhibit poor and variable bioavailability [2,3].

IVIVC is a predictive mathematical model describing the relationship between an in vitro property and a relevant in vivo response. In the case of immediate release dosage forms, the main objective of an IVIVC is to reduce the number of BE studies via supporting the optimization of the drug formulation. A Level A IVIVC is usually established by a two-stage procedure: in vivo absorption is estimated using an appropriate deconvolution technique (e.g., Wagner-Nelson, Loo-Riegelman, numerical deconvolution) followed by the comparison of drug absorbed to the fraction of drug dissolved. Even though the deconvolution method is often applied for regulatory submission, the method is limited to linear pharmacokinetics (PK) regimen [4].

Alternatively, the mechanistic deconvolution using the physiologically based pharmacokinetic (PBPK) modeling popularly known as physiologically based IVIVC (PB-IVIVC) is nowadays extensively utilized for biopharmaceutics modeling [5,6]. Besides its applicability to the nonlinear PK, the PBPK model also considers the different factors governing the drug release and absorption such as particle size of the API, food effect, pH-dependent solubility profile, precipitation, gastric emptying time, drug degradation, drug solubilization in the presence of excess bile acids and permeation across the intestinal membranes [7,8,9,10].

In the present work, we established both PB-IVIVC and conventional (numerical) IVIVC models for the immediate release oral tablet (Xarelto) formulations containing Rivaroxaban (Riva), a BCS II anti-coagulant drug. Riva is reported to exhibit dose-dependent food effects. More precisely, while the lower dose (10 mg) can be taken with or without food, the highest dose strength tablet (20 mg) should be taken with food to attain the positive food effect for oral absorption and systemic availability [6]. As per the regulatory requirement, clinical trials are required to establish bioequivalence between the innovator and generic drug products containing the BCS II drug, and especially exhibiting a food effect. As the critical formulation and drug product information of Riva is still covered by patent protection and no biowaiver exists, the design of a generic formulation containing this drug can be a challenge, especially considering the food effect displayed by the highest dose strength [11,12]. Here, we first developed an in silico PBPK model using the fasted conditions and low-dose formulations. On the basis of developed models, the PK profile of Xarelto formulations of the highest dose strength was predicted in fed conditions. The in vitro permeability of Riva as the pure API alone, and in the reference formulations (Xarelto^®^ IR tablet) was determined using Caco-2 cell lines. Using combined in vitro solubility, dissolution, and permeation data with literature data as the input in the developed PBPK model, the extent of food effect in vivo on the oral absorption of the drug was predicted by mimicking the fed state condition using biorelevant media. In addition, a conventional IVIVC model was established using the two-stage deconvolution method. The dissolution of Xarelto 20 mg IR tablet in USP IV apparatus was determined both in fasted and fed biorelevant media. The correlation was built based on the experimental in vitro dissolution data in fasted media and the mean in vivo plasma concentration profile in the fasted state obtained from the literature. Thereafter, the correlation was used to predict the in vivo profile in the fed state based on the in vitro dissolution in fed biorelevant media. The predicted results of the IVIVC model were compared with the PBPK model. Lastly, a virtual bioequivalence trial was performed to assess the performance of formulation in the fed state taking into consideration the population variability.

## 2. Materials and Methods

### 2.1. Chemical and Reagents

The reference formulations (Xarelto^TM^ of 20 mg strength IR tablet) were acquired from the market. Caco-2 cell line was purchased from American Type Culture Collection (ATCC) (Rockville, MD, USA). Minimal Essential Medium (MEM), fetal bovine serum (FBS), L-glutamine, trypsin (0.25%)-EDTA (1 mM), and penicillin-streptomycin mixture were purchased from Sigma (Vienna, Austria). Simulated fluid powders were purchased from Biorelevant.com Ltd, the UK, for the cell culture studies. Whereas for the solubility and dissolution studies, the simulated media were prepared using sodium taurocholate, lecithin and pepsin purchased from Sigma. The milk used for the solubility studies were purchased from the local market with a natural fat content of 3.5% fat. All other chemicals were of analytical reagent grade.

### 2.2. Software

Simulations were performed using the advanced compartmental absorption and transit model (ACAT) model implemented in the GastroPlus^TM^ (version 9.0., Simulation Plus, Inc., Lancaster, CA, USA). The ACAT model serves as a bridge between the formulation performance and PK parameters of the drug products and hence provides a valuable tool to guide formulation development to achieve the desired quality target product profile. In addition, the simulations from the ACAT models were verified using the conventional IVIVC model developed using the IVIVC Toolkit of Phoenix WinNonlin, Certara, NJ, USA.

### 2.3. Chromatographic Quantitative Analysis

Ultra-High-Performance Liquid Chromatography (UHPLC) was used to quantify Riva in the samples obtained during in vitro dissolution, solubility, and permeability. A Waters Acquity H-Class instrument (Milford, CT, USA) equipped with a PDA detector (operating at a wavelength value of 248 nm) was used. The column used was Acquity UPLC BEH C18, 1.7 µm, 2.1 mm × 50 mm (Waters), and the mobile phase was 3:7 (*v/v*) mixture of acetonitrile and 0.1M Ammonium-acetate buffer. The analysis was performed applying isocratic elution at the flow rate of 0.5 ml min^−1^. The injection volume was 2 µL, and the total run time was 5 min. The dissolved amount of Riva was determined based on the area under the appropriate peak and using external standard calibration.

### 2.4. Biopharmaceutical Properties of Riva

#### 2.4.1. Biorelevant Solubility Determination

Equilibrium solubility of Riva was determined in water, fasted state simulated gastric fluid (FaSSGF), fed state simulated gastric fluid (FeSSGF), fasted state simulated intestinal fluid (FaSSIF), and fed state simulated intestinal fluid (FeSSIF). All the simulated fluids (version 01) were prepared fresh on the day of the experiments conducted. In the case of FaSSIF media, 3 mM and 0.75 mM, and for FeSSIF, 15 mM and 3.75 mM of the sodium taurocholate and lecithin were used, respectively. The FaSSGF media contain 0.08 mM and 0.02 mM sodium taurocholate and lecithin, whereas the FeSSGF media were prepared using an equivalent amount of milk and monobasic sodium phosphate buffer of pH 5. The concentration of different components of the media in detail is mentioned in Table S1 of the Supplementary Materials.

For the solubility determination, an excess amount of the pure drug was added to 5 mL of different simulated fluids. Thereafter, the sample was incubated at RT for 24 h with gentle shaking at 100 rpm using a rotatory shaker. The samples were then filtered using 0.22 µm syringe filter, and the filtrate was analyzed using UHPLC [13,14]. The solubility results of Riva obtained in different simulated media were incorporated in GastroPlus^TM^.

#### 2.4.2. In Vitro Dynamic Biorelevant Dissolution

Dynamic dissolution of Xarelto^TM^ (20 mg) tablets were carried out using USP Apparatus 4 fitted with 12 mm tablet cell using the media mimicking both the fasted and fed gastrointestinal state. In the In Vitro fasted state, the tablet was exposed to FaSSGF (pH 1.6) for 0.5 h, followed by exposure of the same tablet to FaSSIF (pH 6.5) for 5.5 h. The dissolution study was evaluated with 12 replicates. During the dissolution test, samples were withdrawn at predetermined time points and then filtered with 0.7 µm GF/F disc filter (Whatman, Maidstone, UK). The In Vitro fed state experiments were conducted in a similar manner to that of the fasted state, using FeSSGF and FeSSIF as dissolution media. The dissolution media (FaSSGF, FeSSGF, FaSSIF, FeSSIF) were prepared based on the composition available at https://biorelevant.com/shop/ (accessed on 24 February 2021); however, enzymes were excluded and SIF powder was replaced by surfactants such as tween 80 and sodium lauryl sulfate.

#### 2.4.3. In Vitro Caco-2 Permeability Determination

Caco-2 cells (ATCC) were cultured in Minimal Essential Medium (MEM), 20% fetal bovine serum, 2 mM L-glutamine, and 1% penicillin-streptomycin at 37 °C in humid air atmosphere containing 5% CO_2_ in 75 cm^2^ cell culture flasks. Freshly prepared FaSSIF was used for the study.

For the transport studies, 0.5 × 10^6^ cells were seeded per 12-well transwell insert (translucent, 0.4 µm pore size, Greiner Bio-one^®^). Cells were cultured with 500 µL medium in the upper compartment and 1500 µL in the lower compartment. The medium was changed every 2 or 3 days, and transepithelial electrical resistance was measured via EVOM STX-2-electrode (World Precision Instruments, Florida, USA). The cell monolayers were used for the experiments, once the resistance reached a transepithelial electrical resistance (TEER) value of >300 Ω*cm^2^ (18–21 days) [15,16].

Thereafter, the medium was removed, and the cells were coated with 90 µL gastric porcine mucin (40 mg/ml in MEM + 10%FBS) for 30 min. A concentration of 10 µM of Riva and Xarelto^TM^ (20 mg) tablets (equivalent to 10 µM of Riva) was used. For the preparation of Xarelto^TM^ (20 mg) samples, the tablets were ground using a mortar, and pestle and the amount of the formulation containing the desired amount of Riva was dispersed in FaSSIF and stirred for 30 min at RT. The respective suspensions (510 µL) were applied to the upper compartment of the transwell and 1500 µL Krebs Ringer buffer added in the lower compartment. Ten microliters were immediately withdrawn from the apical compartment to determine the total amount applied. Plates with transwell were incubated upon agitation for a total of 120 min. One-hundred-microliter samples were taken from the lower compartment at predetermined time points of 0, 30, 60, 90, and 120 min and replaced by pre-warmed Krebs-Ringer buffer. In addition, at the end of the experiment, 10 µL of the upper compartment were collected for the calculation of the recovery rate. TEER values were measured before and after the transport study to identify potential damage to the cell layer. The permeability of sodium fluorescein (10 µg/mL) in the Krebs-Ringer buffer was determined to verify the barrier properties of the Caco-2 monolayer. All samples were stored at −20 °C until further analysis. On the day of analysis, the samples were thawed at room temperature, and the content was measured using UHPLC.

For the determination of the apparent permeability coefficient (P_app_), the following equation was used, where dQ/dt is the flux across the cell monolayer (ng/s), A the surface of the monolayer (cm^2^), and C the initial concentration in the donor compartment (ng/mL):

(1) $$P_{\mathrm{app}}=\frac{\mathrm{dQ}}{\mathrm{dt}\;\times \;A\;\times \;c}$$

#### 2.4.4. Determination of Systemic Disposition Parameters of Riva

The In Vivo PK profile of Riva after intravenous administration was not found in the literature. Therefore, plasma concentration–time profile after 10 mg oral solution administration under the fasted state was used in the PKPlus^TM^ to obtain the systemic clearance, volume of distribution, half-life, and distribution constants between the central and peripheral compartments [17,18]. Models were fitted empirically in the PKPlus^TM^ employing 1-, 2-, and 3- compartment separately. The Hooke & Jeeves pattern search method was used during the fitting and the weighing was equal to 1/Yhat^2^. Akaike information criterion (AIC) and Schwarz criterion (SC) were used to select the best-fitted compartment model. The obtained PK parameters were then fixed and employed in the simulation of solid oral dosage forms.

### 2.5. Physiologically Based Gastrointestinal Absorption Modeling

#### 2.5.1. Model Compound Parameters

Physiochemical properties of Riva such as molecular weight and lipophilicity were compiled from the literature (Table 1) [19]. Human duodenum effective permeability (P_eff_) was estimated from In Vitro CaCo-2 permeability data using the in-built relation present in the GastroPlus^TM^. The experimentally obtained values of solubility, particle size, and dissolution parameters were used.

#### 2.5.2. Development of In-Silico Physiology Based Gastrointestinal Absorption Model

The ACAT model implemented in GastroPlus^TM^ was used for all the simulations in the current study. Input parameters for the ACAT model can be categorized into three classes, i.e., formulation properties (such as particle size distribution, density, and release profiles of drug products), physicochemical properties of drug substances (such as diffusion coefficient, lipophilicity, pKa, solubility, and permeability), and pharmacokinetic parameters (such as clearance, the volume of distribution, and the disposition model). All other parameters were set at default values in GastroPlus^TM^. The simulations were performed using the default “Human Physiological-Fasted” and “Opt LogD Model SA/V6.1”, in order to simulate the plasma concentration profiles of Riva following oral administration of tablet dose in fed condition. The Opt. LogD SA/V v6.1 model is one of the ACAT models in GastroPlus software. In the ACAT model, the option “CR Dispersed” was selected using the USP4 profile to model the release of the drug. Once the drug is released, solubility and PSD were used to model dissolution of the drug via the Johnson model.

On the basis of LogD value of the drug molecules, the model can automatically fine-tune the absorption scale factor for the different compartment of the intestine, resulting in improved simulation and thus prediction of the regional absorption of the API.

##### Model Verification

Initially, the predictive power, robustness, and the effect of Absorption Scale Factor (ASF) optimization on the absorptive phase of Riva were evaluated. To do so, the predicted pharmacokinetic parameters were compared with the In Vivo data from literature. The pharmacokinetic model was developed using oral solution doses (5 and 10 mg) as well as for oral IR tablet doses (5 and 10 mg) of Riva under the fasted state and compared with the published data [17,18]. The percent prediction error value was calculated to evaluate the accuracy of the model. The calibrated ACAT model for Riva was then applied to simulate the plasma concentration–time profile of Xarelto tablet (20 mg) in the fed state.

##### Parameter Sensitivity Analysis (PSA)

PSA was performed for the uncertain and key parameters in formulations such as mean particle radius, dose volume, particle density, effective permeability, precipitation time, and diffusion coefficient for the dosage forms investigated under the fasted and fed states.

### 2.6. IVIVC Studies

In addition to the PBPK model, the conventional IVIVC was used to predict the PK profile after oral administration of formulation in the fed state using IVIVC Toolkit 8.0 of Phoenix WinNonlin 8.2.0.4383 for Windows (Pharsight, Certara, USA Inc, St. Louis, MO, USA). The IVIVC model was developed and calibrated using the In Vitro dissolution and In Vivo profile of 20 mg strength of Xarelto IR tablet in the fasted condition. Thereafter, using the established IVIVC, the PK profile of the Xarelto IR tablet in fed condition was predicted and compared with the simulation results obtained from the PBPK model.

IVIVC was based on a two-step deconvolution method. Initially, the Weibull function was fitted to the In Vitro data of the Xarelto IR tablet (20 mg tablet) in the fasted condition. Moreover, the time course of In Vivo absorption was derived using deconvolution. The In Vivo data of the 10 mg oral solution published by Kubitza were used as a reference for calculating the unit impulse response (UIR) function [17,18]. The In Vivo data of the PK profiles of the Xarelto IR tablet (20 mg tablet) were then deconvolved and compared to the In Vitro dissolution profiles using the fraction absorbed (F_abs_) vs. the fraction dissolved F_diss_ plot.

Thereafter, in the second step, a correlation was built between the In Vitro drug release and In Vivo drug absorption. The IVIVC model was established based on In Vitro dissolution profile of the Xarelto IR tablet, and In Vivo results were obtained from the literature. As per the condition, the regression slope line most closely aligned with a value of 1.0, whereas the elimination phase of Riva was calculated using the PK profile of 10 mg oral solution.

Thereafter, as the final step, validation of the developed IVIVC model is required, in order to establish quantitative resilience of the predictive capacity of the model. The validation of the model was performed using the results of the In Vivo fate of the formulation used to establish the model, known as internal validation, and/or by the application of a different formulation, known as external validation. In the present model development, the internal validation of the IVIVC model was performed by comparing the predicted and observed PK profile of the reference product Xarelto 20 mg tablet in the fasted condition. Thereafter, the predictability of the model was evaluated using the percentage prediction error (%*PE*) from the following equation:

(2) $$\%PE=100\;\times \;\left(\frac{Predicted\;value-Observed\;value}{Observed\;value}\right)$$

For the development of a robust model, the average absolute *%PE* of ≤10% for the maximum plasma concentration (C_max_) and area under the curve (AUC) establishes the predictability of the IVIVC. Furthermore, the *%PE* for each formulation should not exceed 15%. If the *%PE* conditions are not met for the internal validation, further validation using the external formulation is required [20,21].

### 2.7. Food Effect (FE) Studies of Riva in Simulated Healthy Population

Simulations were carried out for 20 mg dose strength using the fed state physiology of GastroPlus^TM^ to evaluate the quantitative prediction of FE based on the measurements of In Vitro biorelevant solubility and dissolution. The percentage prediction error for the predicted PK parameters was calculated in comparison with the predicted values Xarelto IR tablet (20 mg tablet).

A single-dose (20 mg), three-period virtual trial in 50 subjects was carried out. GastroPlus^TM^ randomly generates subjects by varying physiological factors such as gastrointestinal transit times, pH, fluid volumes, PK parameters as well as compound parameters. Three populations, namely A, B, and C, with 50 subjects each (in order to gain a thorough sampling across all the variables) were given the same treatment (20 mg strength of Xarelto IR tablet).

## 3. Results and Discussion

### 3.1. Biopharmaceutical Properties of Riva

#### 3.1.1. Equilibrium solubility in simulated media

Table 2 depicts the solubility of the Riva in different biorelevant media. The solubility of the Riva was found to be comparable among different media, i.e., water, FaSSGF (pH 1.6), and FaSSIF (pH 6.5). Whereas in the case of FeSSIF and FeSSGF (pH 5.0), the solubility of the Riva was found to be markedly higher as compared to other simulated biorelevant media. The FeSSIF contains 15 mM sodium taurocholate and 3.75 mM lecithin as compared to 3 mM sodium taurocholate and 0.75 mM lecithin in the case of FaSSIF. Thus, an approx. 2-fold increase in the solubility in the Fed conditions could be attributed to the increase in the lipidic component of the media with a higher fraction solubilized in taurocholate and lecithin micelles. The increased solubility of Riva in the Fed state is in accordance with the literature, demonstrating higher bioavailability of equal of more than 80% when taken with food (for 20 mg dose of Riva) [19,22].

#### 3.1.2. In Vitro Release Profile of Riva in Fasted and Fed Conditions

**Figure 1:**
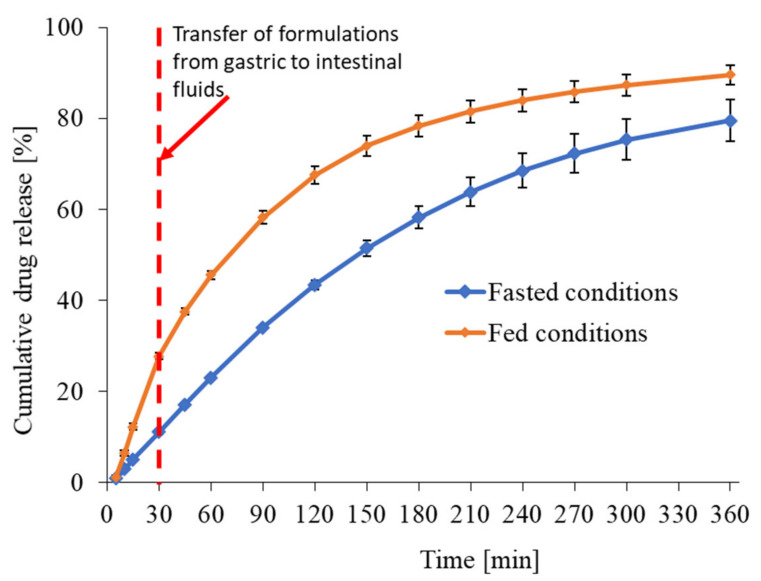
In-vitro release profile of Riva from Xarelto IR tablet (20 mg strength) using USP 4 apparatus. The tablets were transferred from FaSSGF (pH 1.6) and FeSSGF (pH 4.5) media to FaSSIF (pH 6.5) and FeSSIF (pH 5.0) media, respectively, after 30 min.

Figure 1 demonstrates the In Vitro release profiles of the Xarelto IR tablet (20 mg) in fasted and fed conditions using the dynamic dissolution method. After 30 min, In Vitro release of Xarelto IR tablet in the fed state and fasted simulated gastric fluids were found to be 27.7 and 11.0%.

Thereafter, the fed and fasted simulated gastric fluid was replaced via simulated intestinal fluids without removing the Xarelto IR tablets. The In Vitro release, and the time to 80% drug release was found to be the 360 and 210 min in case of fasted and fed state conditions, respectively. Furthermore, the *f*1 (the difference factor) and *f*2 (the similarity factor) were also calculated and found to be 28 and 38, respectively. For bioequivalent In Vitro release profile, the values of *f*1 should be between 0 and 15, whereas the value of *f*2 should be between 50 and 100 [23,24]. Thus, the release profile in the case of the fed condition was found to be significantly higher as compared to the fasted condition. The results demonstrated that the lower solubility in the absence of food components could be the rate-limiting factor for the dose-proportional absorption of Riva from Xarelto IR tablets, independent of the formulation. The significantly higher dissolution in the presence of a food-induced increase in bile salt concentration was found to be in accordance with the solubility study and is the key parameter for the establishment of the PBPK model.

#### 3.1.3. In Vitro Caco-2 Permeability

**Figure 2:**
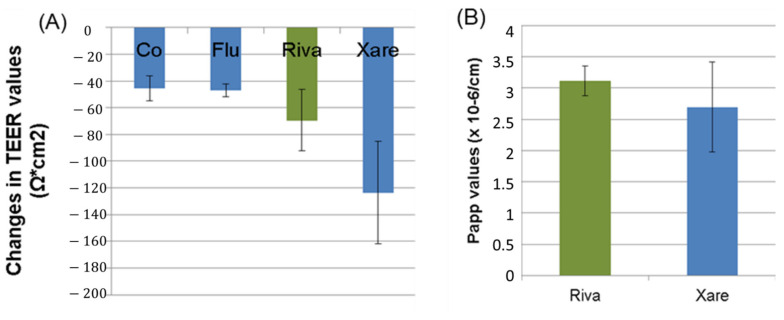
(**A**) Differences in transepithelial electrical resistance (TEER) values between the start value and measurement at the end of the permeation studies. Abbreviations: Control (Co), fluorescein (Flu), rivaroxaban (Riva), Xarelto (Xare). (**B**) Papp values of rivaroxaban (Riva) and Xarelto (Xare).

Permeation of the marker substance, fluorescein, did not cause any alterations of transepithelial electrical resistance (TEER) values compared to controls (no permeation performed). The API powder and, to a greater extent, the formulations caused a decrease in TEER values (Figure 2A). Despite the decrease in TEER values induced by the formulations, there was no increased transport of Riva across the monolayers. Apparent permeability coefficient (P_app_) values (2.69 ± 0.72 × 10^−6^ cm/s) were not increased in the formulations compared to standard Riva (3.11 ± 0.24 × 10^−6^ cm/s, (Figure 2B)). The P_app_ of fluorescein was 0.72 ± 0.13 × 10^−6^ cm/s, indicating a good barrier function of the Caco-2 monolayer and absence of damage by FaSSIF.

P_app_ and transport rates were identical for formulated products and standard Riva. The decrease in TEER values was slightly higher in the formulations than in the unformulated Riva but was not reflected in changes of the P_app_ values. Excipients in the formulations more likely to decrease the TEER values. However, taking into consideration that there is no difference in the P_app_ of pure Riva in comparison with Xarelto (presence of excipients) despite the change in TEER values of CaCo-2 cells in Xarelto, it can be suggested that passage of Riva is mainly transcellular through the CaCo-2 cells. P_app_ values determined in the study were lower than the values published by Gnoth et al. (8.0 ± 0.6 × 10^−6^ cm/s) [25]. The most likely reason for this difference is the lack of mucus production of Caco-2 cells, which can affect permeation. To reproduce the physiological situation, a mucus layer has been added to the Caco-2 monolayer in this study. The effect of mucus on the permeation of active pharmaceutical ingredients (APIs) has been reported controversially [26,27]. The comparison between Caco-2 cells and mucus-producing HT29-MTX did not show prominent differences for many lipophilic and hydrophilic compounds, suggesting that mucus does not represent a strong barrier for the permeation [28]. The exclusive assessment of the role of mucus, however, was not possible because HT29-MTX cells lack P-glycoprotein expression and the lack of reverse transport will increase the measured P_app_ values. Although the same cell types were used, another study reported that the permeability of drugs with a partition coefficient (logP) > 1 was decreased in the mucus-producing cell lines [28]. The passage of Riva might be hindered by mucus because of its logP value of 1.36 [28]. It can be concluded that Riva formulations reacted very similarly and did not display a permeation-enhancing effect on the permeability of Riva. The results obtained of various physicochemical parameters such as those from the solubility study, Caco-2 permeability, and the published literature were used as the input parameters for the development of the PBPK model. The values of the input parameters used in the PBPK model are mentioned in Table 1 [19,29,30,31,32,33].

#### 3.1.4. Systemic Disposition Parameters of Riva

**Figure 3:**
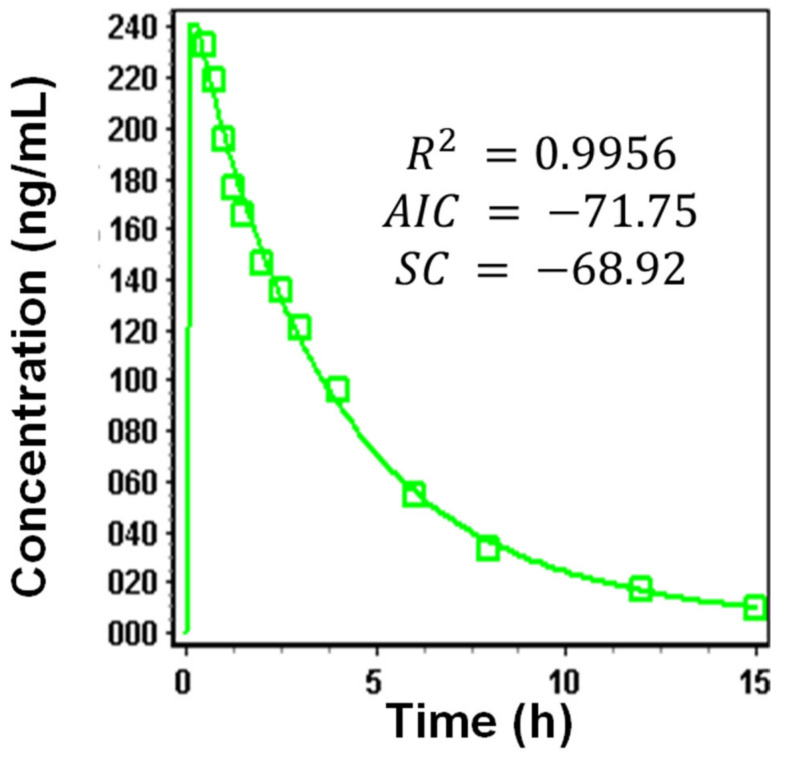
Pharmacokinetic data fitting 2- compartment of oral solution dose (10 mg) of Riva.

The two-compartment model was found to be the best fit model according to the AIC and SC criteria to describe the Riva pharmacokinetics following the administration of oral solution dose (10 mg) (Figure 3). Values of clearance, the volume of distribution (Vc) and, T_1/2_ were in accordance with the literature values. Other parameters such as the peripheral volume of distribution (V_2_), distribution constant from the central to the peripheral compartment (K_12_), and distribution coefficient from the peripheral to central compartment determined (K_21_) from the two-compartment model fitting were used for further simulations. Table 3 depicts the mean baseline values used for the simulation of Riva plasma concentration–time profile following the administration of oral solution and IR formulation doses. The value of clearance and elimination half-life obtained through fitting is found to be in accordance with the value reported in the Xarelto product information after intravenous administration of Riva at a dose of 1 mg [34,35].

### 3.2. Physiology Based Gastrointestinal Absorption Model of Riva Formulation

#### Prediction of PK Profiles and Optimization of ACAT Model

**Figure 4:**
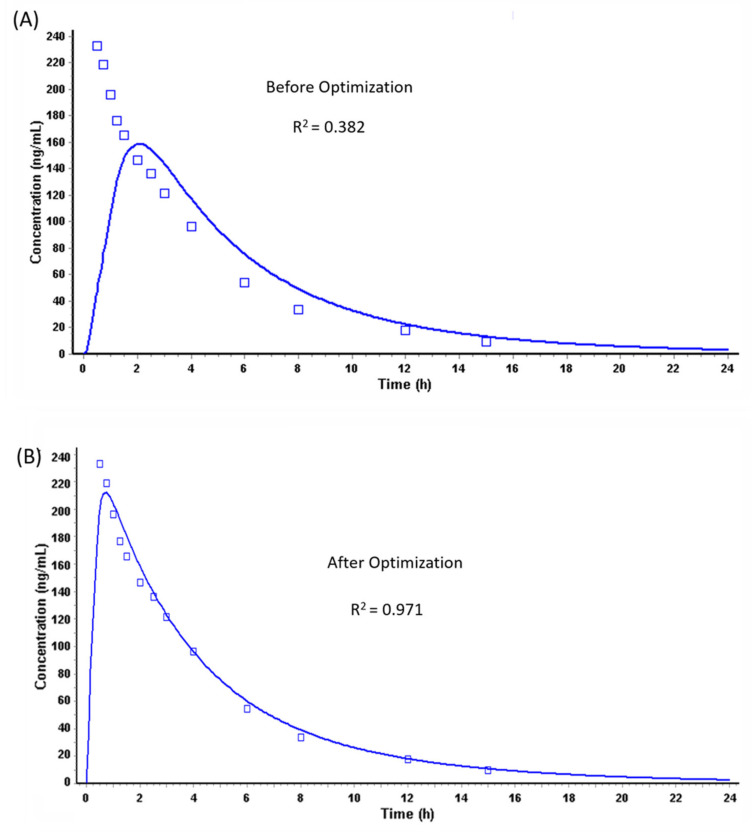
Riva plasma concentration–time profile from solution oral dose (10 mg) (**A**) before and (**B**) after optimization.

Taking into consideration the input parameter as mentioned in Table 1 and the pharmacokinetic parameters mentioned in Table 3, the PK profile of the oral solution dose (10 mg) of Riva was simulated using the ACAT model in GastroPlus^TM^ with default fasted human physiology and ASF values (Table 4). Simulation of 10 mg oral dose with the default Gastroplus^TM^ Human Physiology Fasted largely underestimated the absorption phase of Riva, resulting in the poor fitting of the extracted plasma concentration–time profile obtained for 10 mg oral dose (Figure 4A). This observation suggested that the default fasted human physiology in GastroPlus^TM^ was not able to capture the absorption phase of Riva. Thereafter, the influence of effective permeability (P_eff_) on the C_max_ and T_max_ predictions under fasted conditions for oral solution dose (10 mg) of Riva were evaluated. The results showed that even though the P_eff_ was increased by 10 folds to 3.1, T_max_ was overpredicted by 5 folds and C_max_ was underpredicted by 1.78 folds compared to the average observed values (Table 5) [18].

Furthermore, it is also important to consider the solubility of Riva in gastric medium and concentration of Riva attained with 5 mg and 10 mg of oral solutions. Table 2 reports fasted state gastric solubility of 11 μg/mL (i.e., 0.011 mg/mL) for Riva. Considering 250 mL of dosing volume with instantaneous saturation translates to a solubilization capacity of 2.75 mg in the medium. At a dose of 10 mg, this can generate ~3.6-fold supersaturation, which can lead to faster absorption as reflected by high C_max_ and lower T_max_ in the observed profile (assuming absence of precipitation from the supersaturated state). As simulations were conducted with solubility values of ~11 μg/mL, this could have led to underpredictions.

As a result, the ASF values were optimized using the optimization module in the GastroPlus^TM^, which could capture the absorption phase of the plasma concentration–time profile of oral solution (10 mg) (Figure 4B). ASFs in GastroPlus^TM^ are a multiplier used to scale the effective permeability to account for variations in surface-to-volume ratio, pH effects, influx, or efflux transporter differences, and other absorption-rate-determining effects. On the basis of the GI physiology, ASFs are used to scale the effective permeability of the API across the different sections of the GI tract.

The ASFs were optimized using the PK data set of single-dose oral solution (10 mg) in the fasted state changing the C1 and C2 coefficients of Opt logD Model SA/V 6.1, which determines absorption from the small intestinal compartments. However, the C3 and C4 coefficients which determine absorption from the colon were kept at their default values. The optimized ASF were nearly 14 folds higher than the default values, leading to the faster absorption of Riva in the small intestine. In addition, the default value for compartment volume occupation by water in the colon was reduced from 10% to 2% to better account for measured free water content in the colon [8,36]. All other parameters were set at default values in GastroPlus^TM^; the default and optimized ASF values are mentioned in Table 4.

Table 5 shows the pharmacokinetic parameters obtained from the simulated PK profile of solution oral dose (10 mg) of Riva before and after optimization of ASF and compared with the literature data.

In order to verify that the optimized ASF values for the small intestine can reasonably capture the absorption phase of Riva, simulations of mean plasma concentration–time profile of Riva following administration of oral solution dose (5 mg and 10 mg) and IR tablet formulation (5 mg and 10 mg) under the fasted condition were also carried out. Simulated mean plasma concentration–time profiles of Riva from solution and IR tablet formulation and corresponding pharmacokinetic parameters (C_max_. T_max_ and AUC_0–∞_) calculated are demonstrated in Table 5 and Table 6. All the predictions of pharmacokinetic parameters for different formulation and doses of Riva were within two folds of the reported values. This fosters our confidence in the predictive ability of the developed ACAT model for Riva.

Parameter sensitivity studies were performed investigating the impact of key factors on the bioavailability of Riva. The mean particle size of the API in the IR tablet formulation was found to be the most important factor influencing the bioavailability of Riva (Data not shown) irrespective of fasted and fed state. Other factors such as dose volume, particle density, precipitation time, diffusion coefficient, and P_eff_ seem to have a relatively minor influence on the bioavailability of Riva.

Once the ACAT model was optimized with modified ASF values, pharmacokinetic parameter predictions were carried out for Riva 5 mg oral solution dose (Table 5) and Xarelto IR tablet for 5 mg and 10 mg (Table 6) dose in the fasted condition. The In Vitro release profile and the physicochemical parameters of the API was found to be biopredictive and was able to describe the plasma concentration profiles, and the predicted values of C_max_, AUC, and T_max_ were found to be in agreement with those of the literature, which increased the confidence in the developed ACAT model.

*In silico* simulation of Xarelto (20 mg dose strength) in fasted and fed state

**Figure 5:**
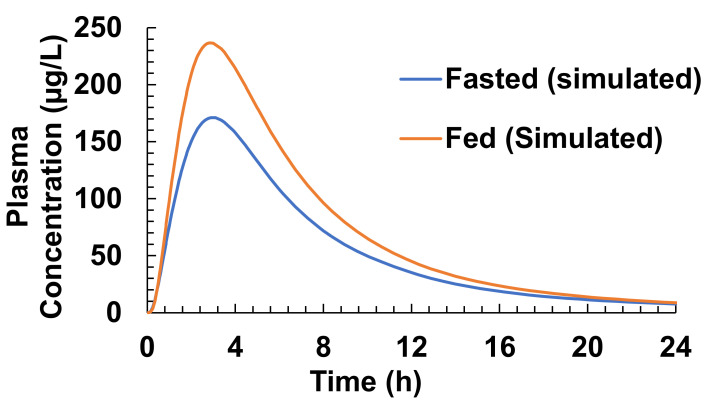
Simulated plasma concentration-time profile of Xarelto (20 mg) in fasted and fed states using GastroPlus^TM^ ACAT model.

The developed ACAT model was used to simulate the plasma concentration–time profile of Xarelto (20 mg dose strength) in the fasted and fed state with the dissolution profiles using the USP 4 flow-through apparatus and biorelevant dissolution medium. It was observed that the inclusion of dissolution profiles of Xarelto IR Tablet (20 mg) during modeling led to an improvement in the simulations with predicted values close to the observed values, as reported in the literature. Table 7 and Figure 5 represent the simulated plasma concentration–time profile and key pharmacokinetic parameters predicted from the simulation of Xarelto (20 mg) IR tablet in the fasted and the fed states.

The results clearly depict the presence of food effects when Xarelto (20 mg) is administered in the fasted and fed states. However, the food effect was a bit underestimated compared to that reported in the literature.

**Figure 6:**
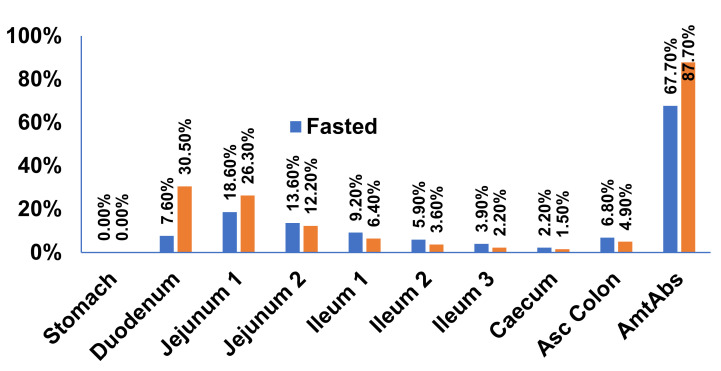
Regional amount absorbed from Xarelto in fasted and fed states in the gastrointestinal tract.

As evident from Figure 6, the increase in bioavailability of Xarelto during the fed state simulation was found, which could be due to the enhanced dissolution of Riva in the fed state. The increase in solubility in the fed state resulted in a greater fraction of Riva to be absorbed from the duodenum and Jejunum 1 as compared to the fasted state. The simulated results are in accordance with the Xarelto product literature outlining site-specific absorption of Riva [34,35].

### 3.3. IVIVC Studies

#### 3.3.1. Modelling

##### In Vitro and In Vivo Raw Data

The In Vitro release profile of Xarelto 20 mg tablet in the fasted state was used for the establishment of the IVIVC model. As evident from the dynamic In Vitro dissolution of the Xarelto (Figure 1), after 30 min of incubation in a simulated gastric medium, the simulated gastric medium was replaced by a simulated intestinal medium. Upon incubating the tablets for 5.5 hours, the amount of API release was found to be approx. 80 and 90% in case of fasted and fed conditions, respectively.

The In Vivo data of the Xarelto 20 mg tablet and 10 mg oral solution in the fasted condition were obtained by literature published by Kubitza and co-workers [17,18]. The T_max_ in the case of solution and tablet was found to be 0.5 and 3 h, respectively. In addition, the C_max_ was reported to be markedly higher in the case of the solution as compared to the tablet. The observation suggests higher absorption of the Riva in presence of solutions, which is reported to be due to the faster and higher amount of Riva available to be absorbed. Thus, suggesting the absorption of Riva to be not limited by permeability.

##### Dissolution Curve Fitting

**Figure 7:**
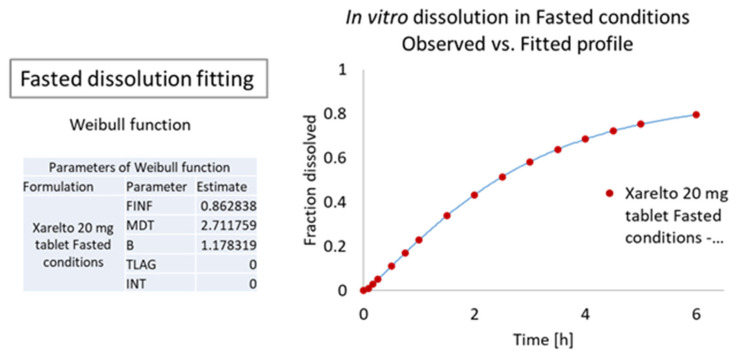
Observed In Vitro profile vs. fitted curves of the Xarelto 20 mg tablet.

The fasted state In Vitro release profile of the Xarelto 20 mg tablet was fitted with the Weibull function. As evident from Figure 7, using the Weibull function, the predicted In Vitro release or, more precisely, the fraction of API dissolved was found to be overlapping with the observed In Vitro release data as a function of time, suggesting that the Weibull function was suitable to fit the dissolution data.

(3) $$\mathrm{Weibull}\;\mathrm{function},\;y\left(t\right)=\;F_{inf}\left[1-e^{-{\left(t/MDT\right)}^{b}}\right]$$

where, *y(t)* or *Fdiss(t)* - Fraction of drug dissolved; dependent variable of the function, *t*: time (h); independent variable of the function, *F_inf_*: Fraction of drug dissolved at infinity time; parameter of the function, *MDT*: mean dissolution time; parameter of the function, *b*: beta; shape parameter of the function

#### 3.3.2. Calculation of Unit Impulse Response (UIR) Function

**Figure 8:**
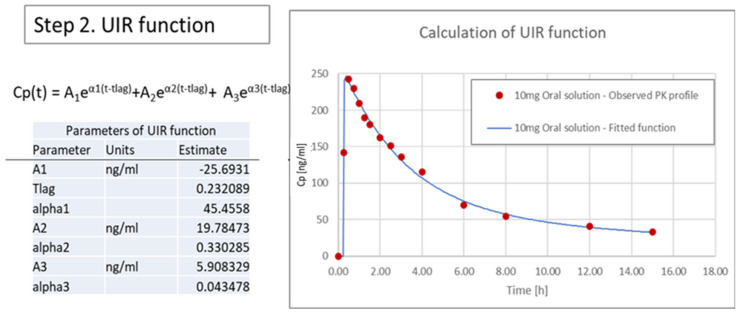
Properties of the unit impulse response (UIR) function.

**Figure 9:**
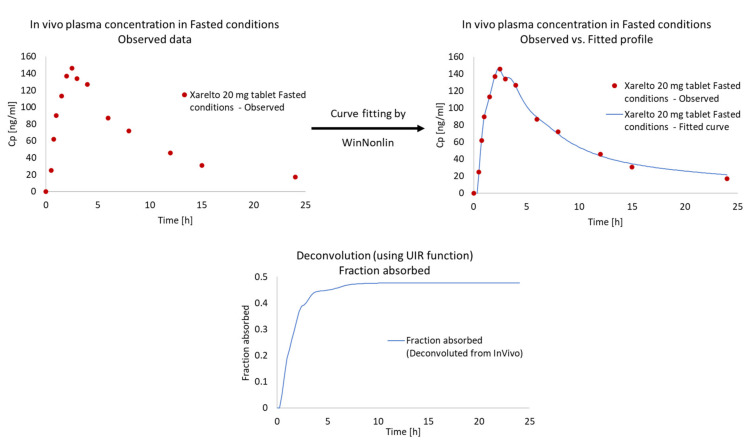
Calculated absorption after deconvolution of Xarelto 20 mg tablet.

To obtain the In Vivo data, the pharmacokinetic parameters of 10 mg oral solution was used. Thus, the UIR function was used to calculate the oral pharmacokinetic data. As evident from Figure 8, using the UIR function, the observed pharmacokinetic profile was found to be overlapping with the predicted pharmacokinetic profile and a linear relation was established suggesting the best fit model. The UIR function obtained were then used to deconvolute the In Vivo pharmacokinetic profile of Xarelto 20 mg tablet in fasted condition, for the assessment of In Vivo absorption profile (Figure 9).

UIR Function, Cp(t) = A_1_e^α1(t-tlag)^ + A_2_e^α2(t-tlag)^ + A_3_e^α3(t-tlag)^ (4)

Where, Cp(t): Concentration of drug in plasma [ng/L]; dependent variable of the function, t: time [h]; independent variable of the function, A (coefficient) and α (exponential); parameter values, tlag: lagtime; parameter value.

#### 3.3.3. Correlation

**Figure 10:**
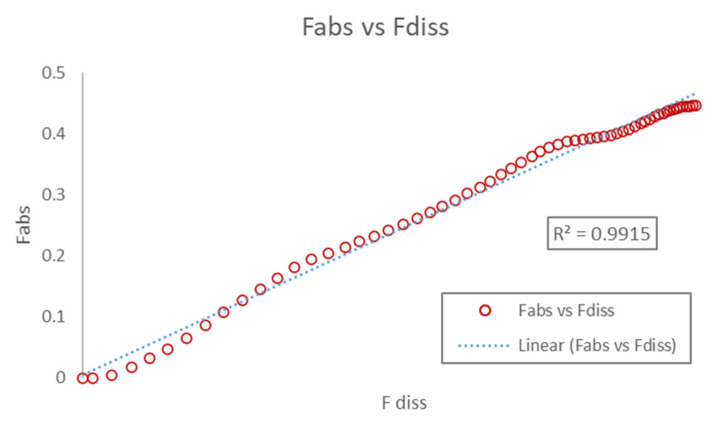
Fraction absorbed In Vivo vs. fraction dissolved In Vitro for the Xarelto 20 mg tablet in the fasted condition.

The dissolved API fraction (obtained from the fitting of In Vitro release profile) was then related with the absorbed In Vivo fractions (obtained from the deconvolution of plasma concentration). As evident from Figure 10, the In Vivo fraction absorbed was found to be linearly correlated with the fraction of API dissolved In Vitro. The observed relation was found to be aligned with the linear regression analysis. Thus, the slope and regression values of Fabs vs. Fdiss plot suggest the development of a robust mathematical IVIVC model.

Correlation equation, F_abs_ = AbsScalexDiss(T_scale_ × T_vivo_ − T_shift_) (5)

where, Fabs: Fraction of drug absorbed; dependent variable of the function, Tvivo: In Vivo time (h); independent variable of the function, AbsScale: Absorption scale factor, T_scale_: Time scaling factor, T_shift_: Time scale shift, Diss: Internal function that linearly interpolates the predicted dissolution data, i.e., the predicted data from the dissolution model fitted to the In Vitro data.

The AbsScale, Tscale, and Tshift parameters of the correlation equation were found to be 0.595, 1.494, and 0.188, respectively.

#### 3.3.4. Internal Validation and Prediction

**Figure 11:**
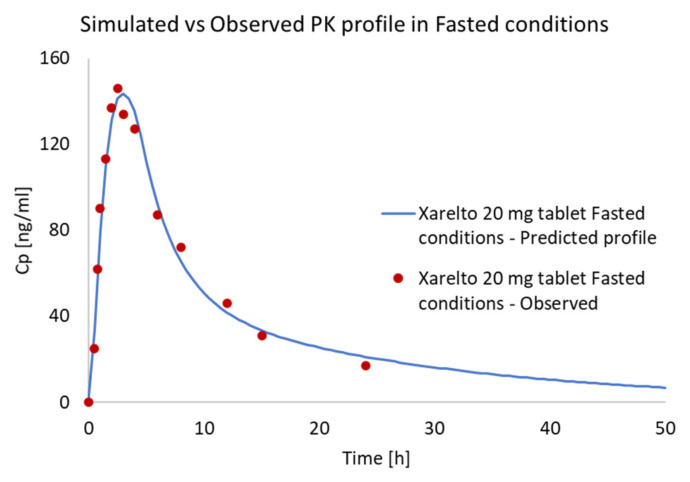
Internal validation (Xarelto 20 mg tablet Fasted condition) of the In Vitro–In Vivo correlation (IVIVC) model.

An IVIVC model was established based on the USP IV fasted state dissolution data and using published In Vivo data as an internal validation of the reference product Xarelto 20 mg tablet (Figure 11). The result of the internal validation was promising (<2% error).

**Figure 12:**
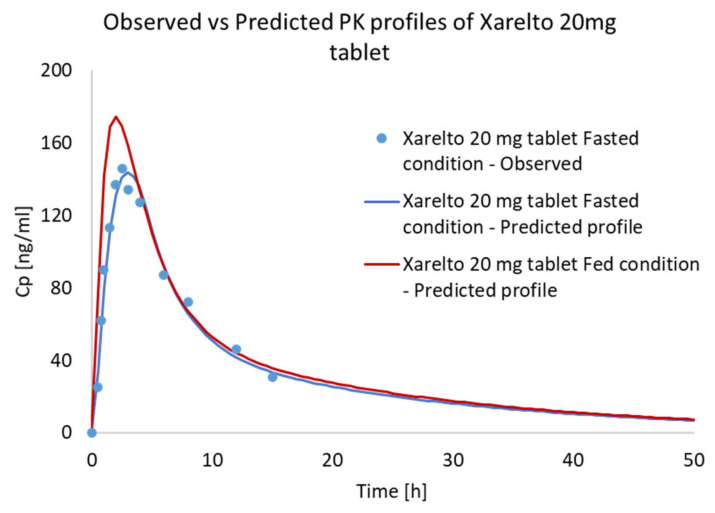
Predicted mean In Vivo profiles of Xarelto 20 mg tablet using IVIVC model.

The IVIVC model was then used for the prediction of In Vivo performance of the Xarelto 20 mg tablet in the fed state. As evident from Figure 12 and Table 8, the C_max_ and AUC were found to be distinctly higher, whereas no significant difference in the T_max_ was predicted in the case of the fed state as compared to the fasted condition. The increase in absorption was found to be in accordance with the increase in solubility of Riva in the presence of simulated fed media. Thus, the developed IVIVC was able to adapt the effect of fed media, predicting the In Vivo profile in the fed state. The developed IVIVC model (developed for reference product) can also be used as an effective tool to predict the In Vivo fate of formulations with different strength and release profiles, considering an average percentage error of less than 10% and tolerance limit in the range of 0.8–1.25.

In the present report, both PBPK absorption and IVIVC model were developed to predict the food effect on the In Vivo fate of the Riva released from Xarelto 20 mg tablet. Both models predicted a higher amount of Riva absorption in case of fed conditions, which could be due to higher solubility and release profile in simulated fed media [17]. The findings are in accordance with the literature, which reported an increase in Riva AUC and the mean C_max_ by 39% and 76%, respectively, when the 20 mg tablet was orally administered with food [37]. Interestingly, both 10 and 20 mg strength of the marketed formulation contain sodium lauryl sulfate (SLS) in order to increase the solubility of the API in the In Vivo conditions. However, as evident from the lower bioavailability, the solubilization efficiency of SLS in increasing the solubility of Riva in the 20 mg tablet was found to be less as compared to the Riva in 10 mg tablet [37]. This decrease in solubilization efficiency in the fasted state could be due to a higher amount of API above the saturation solubility in the case of 20 mg Riva, as the volume of the In Vivo fluid remains the same, potentially resulting in local precipitation and thus reduced bioavailability. Now in the case of the fed state, the excess of API higher than the saturation solubility could dissolve in lipidic components of the food and thus resulted in higher AUC and mean C_max_. On further increasing the dose to supra-therapeutic levels of 50 mg of Riva, no further increase in the AUC and mean C_max_ values was observed even in the presence of fed conditions [33,37]. The ceiling effect observed in the case of 50 mg of Riva dosing could be due to attainment of maximum solubility in fed conditions, assuming the volume of food is nearly alike.

The C_max_ and AUC values predicted by the PBPK and IVIVC model were found to be comparable. However, the C_max_ and AUC values in the case of the PBPK model were slightly higher compared to the IVIVC model. The increase in C_max_ and AUC values in the case of the PBPK model could be due to the incorporation of different physicochemical and formulation properties, for the development of the model, whereas the IVIVC model lacks integration of such parameters. In the present case, as evident from the parameter sensitivity studies, the particle size of the API was found to be a critical factor affecting the release profile and thus the bioavailability of the Riva. The impact of particle size on the bioavailability was found to be in accordance with the product filling, as the reference formulation was developed using the micronized Riva in order to improve oral bioavailability via increasing solubility [37]. Thus, the development/optimization of the PBPK model using the particle size distribution of API could be responsible for the slight difference in the predicted C_max_ and AUC values compared to the IVIVC model. Thus, the developed model can further be used to develop and optimize the formulation parameters, mainly in the early stage development phase, reducing preclinical and clinical time and cost.

### 3.4. Food Effect (FE) Studies of Riva in Simulated Healthy Subjects

**Figure 13:**
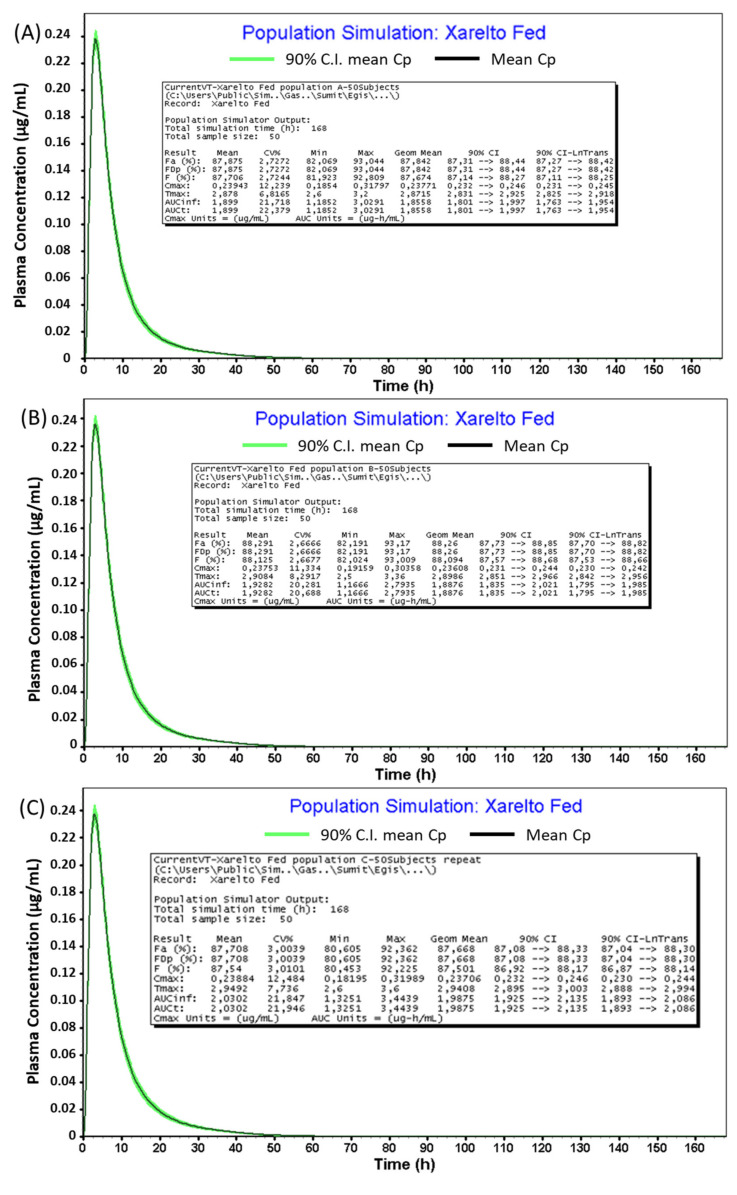
Virtual In Vivo pharmacokinetic profile of Xarelto IR tablet (20 mg Strength) for Populations (**A**–**C**) in fed conditions.

A virtual trial is a stochastic simulation that randomly samples parameters from predefined distributions. In order to take into account the effect of population variability on the plasma concentration profile of Riva following administration of Xarelto, a single dose (20 mg) 3-period virtual study design was carried out in the fed state. Three virtual populations of 30-year American Male/Female A, B, and C, each of 50 subjects, were created and subjected to Xarelto IR tablet (20 mg tablet). GastroPlus^TM^ randomly generates subjects by varying the physiological factors such as gastrointestinal transit times, pHs, fluid volumes, and pharmacokinetics parameters, as well as compound parameters [38]. Table 9 and Figure 13 provides a summary of in silico investigation of food effect variability across virtual populations.

The predicted population In Vivo pharmacokinetic profile of Xarelto IR tablet (20 mg strength) in the fed condition was found to be comparable in populations A, B, and C in the conditions, i.e., the average value of population geometric means were found to be within the range of 80–125% compared to the mean predicted In Vivo profile mentioned in in Table 7, Figure 5 (C_max_ and AUC_0–∞_ value of 236.64 and 1857.3, respectively). However, marked difference (>80–125%) was observed in case of fasted conditions (C_max_ and AUC_0–∞_ value of 171.15 and 1433.8, respectively), as compared to the predicted In Vivo population pharmacokinetics of Xarelto IR tablet (20 mg strength) in fed conditions.

During the virtual simulations, the physiological and pharmacokinetic parameters of the same subjects were identical for reference formulations. However, in reality, physiological and pharmacokinetic parameters could fluctuate within the same subjects if they were given different formulations on different occasions. Thus, by incorporating this intra-subject variability, it is possible that In Vivo profile of Xarelto IR tablet in the fasted condition might be bioequivalent to the population kinetics of Xarelto IR tablet (Fed) in population A since its 80% confidence interval for the AUC value (77%) is close to the edge of the BE limits.

## 4. Conclusions

In the present manuscript, a mechanistic physiology-based model for the Xarelto IR tablet was developed considering different physicochemical and pharmacokinetic parameters. In addition, a conventional IVIVC model was also developed in order to verify the In Vivo profile obtained via the PBPK model. The validation results demonstrated the development of successful models, and the predicted In Vivo profiles from both models were found to be comparable. The results demonstrated a significant food effect increasing the C_max_ of the Riva, which could be due to higher solubility in Fed conditions. The developed model strategy can be effectively adopted to increase the confidence of the model. Furthermore, the PBPK model can also lead to the establishment of the biased dissolution methods crucial for the generic company to establish bioequivalence mainly focusing on new formulations with a similar drug release mechanism using external validation.

## Figures and Tables

**Table 1:** Input parameters of Riva for building the PBPK model in GastroPlus^TM.^

Physiochemical Parameter	Values
Molecular Weight (g/mol)	435.89
logP	1.36
pKa	strongest acidic: 13.6strongest basic: 1.6
Solubility vs. pH	water solubility (pH = 7) = 10 µg/mL pH 1.2, FaSSGF = 11 µg/mL pH 6.5, FaSSIF = 9.9 µg/ml pH 5.0, FeSSIF =16.8 µg/ml
Particle Size (Radius)	7.5 µm (Xarelto tablet, 20 mg) d_90_ = 9.4 µm; d_50_ =3.8 µm; d_10_ =0.7 µm
Caco-2 Permeability Dissolution Profiles (USP 4)	2.69 ± 0.72 × 10^−6^ cm/s (Xarelto) Xarelto IR tablet (20 mg)

**Table 2:** Solubility of Riva in biorelevant media.

Solubility in	pH	Values (µg/mL)
Unbuffered water	7.0	10.0
FaSSGF	1.6	11.0
FaSSIF	6.5	9.9
FeSSGF	4.5	24.0
FeSSIF	5.0	16.8

**Table 3:** PK parameters obtained from two-compartment model fitting of oral solution (10 mg) of Riva.

Parameter	Values
Clearance (L/h)	9.43
V_c_ (L/kg)	0.47
T_1/2_ (h)	4.62
K_12_ (1/h)	0.04
K_21_ (1/h)	0.21
V_2_ (L/kg)	0.09

**Table 4:** ASF values before and after optimization of ACAT model.

Compartment	Default (GastroPlus) ASF	Optimized ASF
Stomach	0	0
Duodenum	2.673	36.44
Jejunum 1	2.658	36.25
Jejunum 2	2.629	35.85
Ileum 1	2.592	35.35
Ileum 2	2.568	35.02
Ileum 3	2.505	34.16
Caecum	0.535	0.535
Asc Colon	1.038	1.038

**Table 5:** PK parameters obtained from simulated PK profiles.

Parameter	Actual (Reported) ^a^	Predicted (Before Optimization)	Predicted (After Optimization)
PK parameters obtained from simulated PK profile of solution oral dose (10 mg) of Riva before and after optimization			
C_max_ (µg/L)	266/25.1 (187–412)	159.27	212.23
T_max_ (h)	0.50 (0.25–1.00)	1.92	0.72
AUC (µg·h/L)	997/25.1 (613–1383)	1056	1058
F%	>90%	99.54	99.79
Pharmacokinetic parameters for 5 mg oral (solution) dose of Riva in fasted conditions			
C_max_ (µg/L)	119/18.5 (97.2–158)	80.13	107.06
T_max_ (h)	0.63 (0.5–0.75)	1.92	0.66
AUC (µg·h/L)	461/17.2 (348–587)	528.24	529.37
F%	>90%	99.58	99.80

^a^—taken from literature; data are represented as geometric means/percent geometric coefficient of variation and range in case of reported data [18].

**Table 6:** Pharmacokinetic parameters for IR Tablet in fasted conditions.

Parameter	Actual (Reported) ^a^	Predicted (Optimized ASF)
Pharmacokinetic parameters for 5 mg oral (IR Tablet) dose of Riva in fasted conditions		
C_max_ (µg/L)	72/19.7 (55–96)	76.13
T_max_ (h)	1.88 (0.5–4.00)	2.1
AUC (µg·h/L)	466/23.0 (348–677)	524.42
F%	80–100%	98.86
Pharmacokinetic parameters for 10 mg oral (IR Tablet) dose of Riva in fasted conditions		
C_max_ (µg/L)	141/15.5 (112–184)	149
T_max_ (h)	2.00 (0.5–2.50)	2.20
AUC (µg·h/L)	1020/14.9 (797–1217)	1037
F%	80–100%	97.75

^a^—taken from literature; data are represented as geometric means/percent geometric coefficient of variation and range in case of reported data [18].

**Table 7:** Key pharmacokinetic parameters predicted from the simulation of Xarelto (20 mg) IR tablet in the fasted and fed states.

Parameters	Fasted State		Fed State	
	Actual (Reported) a	Predicted (Optimized ASF)	Actual(Reported) b	Predicted (Optimized ASF)
Cmax (µg/L)	173/35.6 (111–294)	171.15	294.4/15 (225.4–360.6)	236.64
Tmax (h)	1.50 (0.5–4.00)	3	3.00 (0.5–6.00)	2.9
AUC (µg·h/L)	1612/36.1 (859–2193)	1433.8	2294/19 (1464–3227)	1857.3
F%	66%	67.57	80–100%	87.54

^a^—taken from Reference [18]; b—taken from Reference [17]; data are represented as geometric means/percent geometric coefficient of variation and range in case of reported data.

**Table 8:** Observed and predicted values of the internal validation (Xarelto 20 mg tablet fasted condition) and predicted values of theXarelto 20 mg tablet fed condition.

Formulation	Parameter	Predicted	Observed ^a^	%PE	Ratio
Xarelto 20 mg tablet Fasted condition	AUClast (µg·h/L)	1381.946	1361.125	1.52966	1.015297
Xarelto 20 mg tablet Fasted condition	Cmax (µg/L)	143.567	146.000	−1.66657	0.983334
Xarelto 20 mg tablet Fasted condition	Tmax (h)	3.0	1.5	N/A	N/A
Xarelto 20 mg tablet Fed condition	AUClast (µg·h/L)	1543.120	1750.175	13.41790	1.134179
Xarelto 20 mg tablet Fed condition	Cmax (µg/L)	174.566	241.000	38.05670	1.380567
Xarelto 20 mg tablet Fed condition	Tmax (h)	2.0	3.0	N/A	N/A

^a^—taken from References [18,17].

**Table 9:** Summary In Vivo pharmacokinetic profile of Xarelto IR tablet (20 mg Strength) for Populations A, B, and C in fed conditions.

Parameters	Population A	Population B	Population C
C_max_ (ng/mL)	238	236	237
AUC_0–__∞_ (ng·h/mL)	1856	1888	1988

## Data Availability

The data presented in this study are available in the research article and supplementary material here.

## Supplementary Materials

The following are available online at https://www.mdpi.com/1999-4923/13/2/283/s1, Table S1: Concentration of different components of the simulated media used in the solubility and dissolution studies, Figure S1: Predicted combined effect of Riva particle size and dose on the bioavailability.

## Abbreviations:

Absorption Scale Factors	ASF
Active pharmaceutical ingredients	APIs
Advanced compartmental absorption and transit	ACAT
Akaike information criterion	AIC
Apparent permeability coefficient	Papp
American Type Culture Collection	ATCC
Bioavailability	BA
Bioequivalence	BE
Biopharmaceutical Classification System	BCS
Immediate release	IR
In Vitro–In Vivo correlation	IVIVC
Minimal Essential Medium	MEM
Fasted state simulated gastric fluid	FaSSGF
Fasted state simulated intestinal fluid	FaSSIF
Fed state simulated gastric fluid	FeSSGF
Fed state simulated intestinal fluid	FeSSIF
Fetal bovine serum	FBS
Parameter Sensitivity Analysis	PSA
Pharmacokinetics	PK
Physiologically based pharmacokinetic	PBPK
Rivaroxaban	Riva
Schwarz criterion	SC
Sodium lauryl sulfate	SLS
Transepithelial electrical resistance	TEER
Ultra-High-Performance Liquid Chromatography	UHPLC
Unit impulse response	UIR
United States Pharmacopeia	USP
