# Supplementary material for: On Absorption Modeling and Food Effect Prediction of Rivaroxaban, a BCS II Drug Orally Administered as an Immediate-Release Tablet
Source: Pharmaceutics. 2021 Feb 20;13(2):283. doi: 10.3390/pharmaceutics13020283 (PMC7923293; doi:10.3390/pharmaceutics13020283)
Supplement: Supplementary file 1 [file pharmaceutics-13-00283-s001.pdf]

pharmaceutics

Supplementary Materials: On Absorption Modeling and Food Effect Prediction of Rivaroxaban, a BCS II Drug Orally Administered as an Immediate-Release Tablet

Varun Kushwah, Sumit Arora, Miklós Tamás Katona, Dattatray Modhave, Eleonore Fröhlich, Amrit Paudel

Table S1. Concentration of different components of the simulated media used in the solubility and dissolution studies.

	FaSSGF	FaSSIF	FeSSGF	FeSSIF
PH	1.6	6.5	5.0	5.0
Solubility [µg/ml]	11.0	9.9	24.0	16.8
Sodium taurocholate (mM)	0.08	3.0	-	15
Lecithin (mM)	0.02	0.75	-	3.75
Sodium chloride (mM)	34.2	105.9	237.02	203.2
Sodium hydroxide (mM)	-	8.7	-	101
Hydrochloric acid (mM)	25.1	-	-	-
Monobasic sodium phospate (mM)	-	28.4	-	-
Acetic acid (mM)	-	-	17.12	144.1
Pepsin (mg/ml)	0.1	-	-	-
Sodium acetate anhydrous (mM)	-	-	29.75	-
Milk/Buffer	-	-	1:1	-

Results of sensitivity analysis: PSA analysis clearly showed that the bioavailability of Rivaroxaban is influenced by both the dose as well as the particle size of the pure API. Effect on particle size on bioavailability is more pronounced at higher dose strength

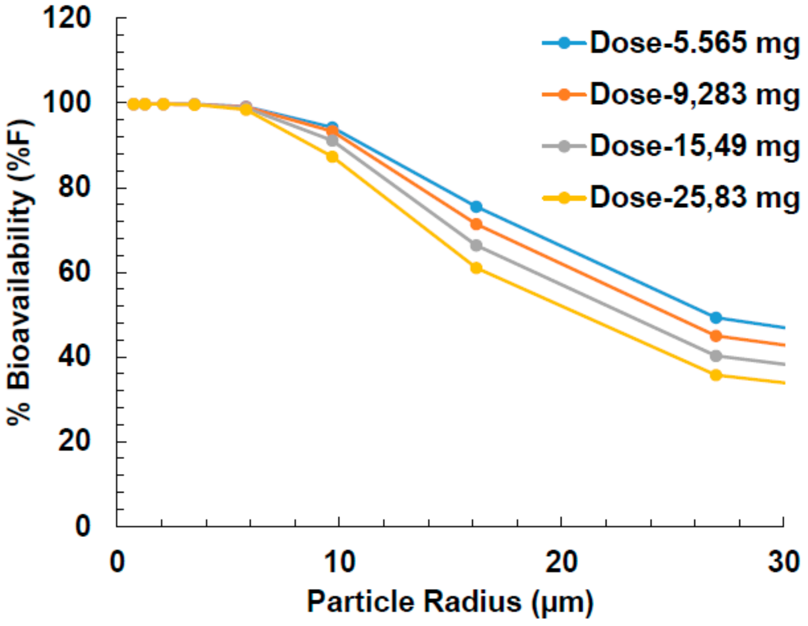

Figure S1. Predicted combined effect of Riva particle size and dose on the bioavailability.
